# Fluorescence-tunable Ag-DNA biosensor with tailored cytotoxicity for live-cell applications

**DOI:** 10.1038/srep37897

**Published:** 2016-11-30

**Authors:** Nelli Bossert, Donny de Bruin, Maria Götz, Dirk Bouwmeester, Doris Heinrich

**Affiliations:** 1 Leiden Institute of Physics, LION, Huygens-Kamerlingh Onnes Laboratory, Leiden University, The Netherlands; 2 Fraunhofer-Institut for Silicate Research ISC, Würzburg, Germany; 3 Fakultaet fuer Chemie und Pharmazie, Julius-Maximilians-Universitaet Würzburg, Germany; 4 Physics Department and California Nanosystems Institute UCSB, Santa Barbara, USA

## Abstract

DNA-stabilized silver clusters (Ag-DNA) show excellent promise as a multi-functional nanoagent for molecular investigations in living cells. The unique properties of these fluorescent nanomaterials allow for intracellular optical sensors with tunable cytotoxicity based on simple modifications of the DNA sequences. Three Ag-DNA nanoagent designs are investigated, exhibiting optical responses to the intracellular environments and sensing-capability of ions, functional inside living cells. Their sequence-dependent fluorescence responses inside living cells include (1) a strong splitting of the fluorescence peak for a DNA hairpin construct, (2) an excitation and emission shift of up to 120 nm for a single-stranded DNA construct, and (3) a sequence robust in fluorescence properties. Additionally, the cytotoxicity of these Ag-DNA constructs is tunable, ranging from highly cytotoxic to biocompatible Ag-DNA, independent of their optical sensing capability. Thus, Ag-DNA represents a versatile live-cell nanoagent addressable towards anti-cancer, patient-specific and anti-bacterial applications.

DNA-encapsulated silver nanoclusters (Ag-DNA) have recently attracted much attention, as they exhibit numerous unique fluorescence properties, like high photostability, fluorescence quantum yields of up to 90% and tunable excitation and emission spectra^1^,^2^,^3^,^4^,^5^,^6^. The high sensitivity to their surroundings is one of the most intriguing properties of Ag-DNA nanoclusters, allowing them to exhibit an optical response due to environmental changes: conformational changes of the DNA template lead to varying distances and interactions between DNA bases and silver clusters^7^,^8^. In solution, Ag-DNA have been shown to even respond to single-base mutations^9^,^10^, and to detect molecule concentrations down to 0.37 nM^11^. Therefore, Ag-DNA are very promising for intracellular bio-sensing applications and to investigate intracellular dynamics, as so far is done mostly with fluorescent nanobead probes^12^,^13^,^14^,^15^. Most fluorescent agents show functional limitations, like photobleaching or low absorption coefficients and often fulfill one function only. This makes the search for alternative nanomaterials an ongoing quest.

The encapsulated silver in Ag-DNA nanoagents further allow for multifunctional applications, as these optical sensors exhibit tunable degrees of cytotoxicity^16^. In the past years, silver has been increasingly used in cosmetics, clothes, implant coatings, and for burn wound dressings, to obtain an antibacterial effect^17^,^18^,^19^,^20^. Its inherent toxicity makes the metal a particularly promising alternative to antibiotics, as bacterial resistances rise^21^,^22^, as well as an anti-cancer agent^23^,^24^,^25^. However, the toxicity mechanism of silver is not yet clearly understood^26^,^27^. As nano-sized silver is highly bioactive, its broad utilization bears a potential risk for humans^28^,^29^,^30^, making a more controlled and tunable way of its application appealing.

Furthermore, these fluorescent Ag-DNA nanomaterials are easily compatible with DNA nanotechnology and DNA origami, and provide an easy attachment site for specific addition of desired targets and recognition elements. This has been implemented for specific labeling of cellular membrane and nuclei^31^,^32^,^33^,^34^, using the Ag-DNA emitters as a replacement of an organic dye. So far, however, the optical sensor functionality, particularly in combination with independently tunable cytotoxicity of Ag-DNA constructs, has not been investigated in live-cell interiors.

Fluorescent Ag-DNA are synthesized upon reduction of silver ions by sodium borohydride (NaBH_4_) in the presence of DNA (Fig. 1a), resulting in rod-shaped silver cores comprised of 4–12 neutral Ag atoms^35^,^36^. Remarkably, these nanomaterials can exhibit peak emission wavelengths across the visible and near-IR spectrum^37^ determined by the variant sizes and aspect ratios of the noble metal nanoclusters^36^. Similar to larger noble metal nanoparticles, larger silver rods correspond to a longer wavelength resonance than smaller particles^38^,^39^,^40^, with the DNA template also playing a role in the fluorescence variance of the emitters.

Using these dependencies, we designed Ag-DNA templates to function as an optical sensor in living cells’ interior environments (Fig. 1b–d) (for DNA sequences please see Table S1). At the same time, by utilizing the endogenous silver core, we tuned Ag-DNA from cytotoxic to completely biocompatible (Fig. 1b–d), creating an intracellular toxicity-tunable optical sensor. Here, we demonstrate the functionality of the designed nanoagents in a live-cell environment.

## Results and Discussion

### Ag-DNA nanoagent design

The design of DNA sequences is the result of optimizing fluorescence and/or sensor properties of Ag-DNA within the cell by varying the base sequences for enhanced performance. The strong interaction of cytosine and guanine bases with Ag(I)ions allows effective stabilization of a silver cluster. Furthermore, the addition of adenine and thymine bases throughout the DNA strand increase the yields of fluorescent species and fluorescence quantum yields. By varying the base sequences, the resulting different sizes and shapes of Ag-DNA structures define their particular functionality as nanoagents.

**Figure 1:**
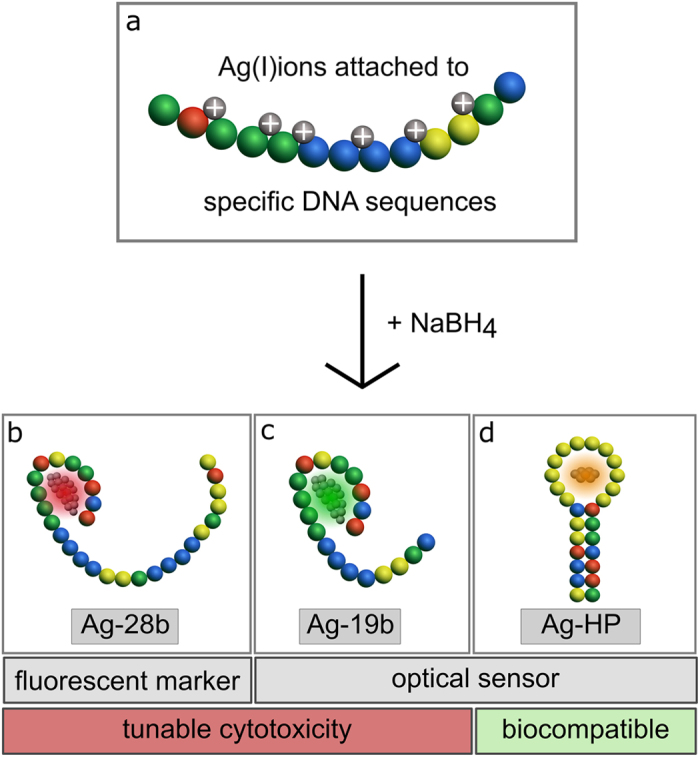
Schemes of general Ag-DNA synthesis (**a**) and specifically designed Ag-DNA constructs (**b–d**). (**a**) Fluorescent Ag-DNA are synthesized upon reduction of silver ions by sodium borohydride (NaBH_4_) in the presence of a specific DNA template. The designed Ag-DNA structures, utilized here, show different functionalities as a fluorescent marker or as an optical sensor, as well as exhibit tunable cytotoxicity or biocompatibility. DNA sequences color code are displayed for (**b**) Ag-28b, (**c**) Ag-19b and (**d**) Ag-HP by Cytosine = yellow, Guanine = green, Thymine = blue, Adenine = red.

First, we demonstrate a robust and bright Ag-DNA structure, applicable as a fluorescent marker inside living cells. The DNA template, consisting of 28 single bases, is designed to yield an Ag-DNA construct (Ag-28b in Fig. 1b) with strong and stable fluorescence properties upon insertion into living cells. Thus, in this DNA-design, the encapsulated silver cluster preserves its fluorescence properties when inside living cells, not reacting to the complex intracellular environment.

While the relatively long single strand wrapping around the silver cluster seems to protect the cluster enough to maintain a strong fluorescence, any exposed parts of the silver surface could still interact with cellular molecules, altering cellular processes.

Small changes in the DNA template can have a significant effect on the functionality of the Ag-DNA nanomaterials. Here, we demonstrate that by a simple modification of Ag-28b, we produced an Ag-DNA construct which shows optical sensor functionality inside living cells: shortening the 28b sequence by nine bases produces the Ag-19b construct (Fig. 1c), which exhibits an optical response to the intracellular environment. By using the shorter sequence, we reduce the shielding of cellular molecules from the silver surface as compared to Ag-28b, resulting in a higher level of cytotoxicity and yielding a stronger sensitivity to the change in conditions inside living cells.To decrease the toxicity level to a biocompatible Ag-DNA for an optical biosensor sensitive to intracellular ions, we designed an Ag-hairpin (Ag-HP) construct (Fig. 1d). As the molecular dynamics of single- and double-stranded DNA heavily depend on the presence of salts^42^, DNA-ions interactions lead to conformational changes of the hairpin loop resulting in optical emission shifts inside living cells, yielding an optical sensor. The amount of cytosine bases in the loop, and the length and sequence of the double stranded stem were optimized for sensitivity. The Poly-C-loop wrapping around the silver cluster through strong Cytosine-Ag(I)ions interactions prevents the silver surface from interacting with cellular molecules, yielding a biocompatible construct.

### Fluorescence-tunable properties of Ag-DNA optical sensors

**Figure 2:**
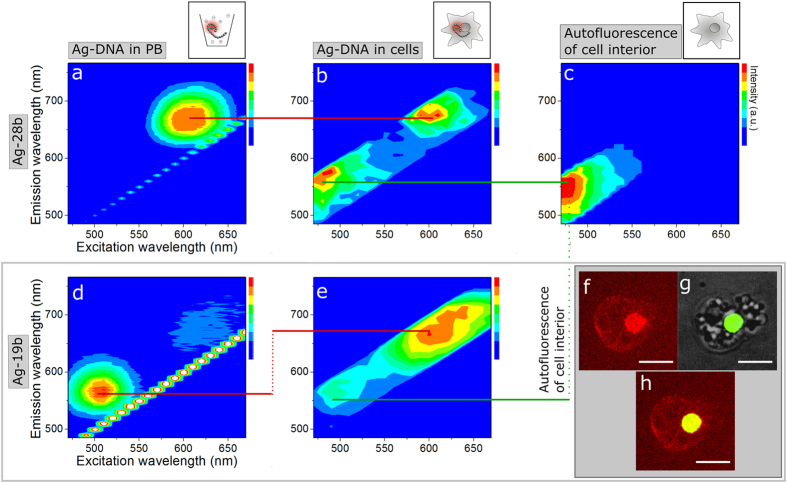
Fluorescence excitation/emission spectra of Ag-DNA nanoagents of type Ag-28b and Ag-19b, before (**a,d**) and after (**b,e**) internalization in *D. discoideum* wildtype cells. Fluorescence spectra are measured within the excitation range of 470–670 nm and the emission range of 490–760 nm, each in 10 nm steps. Ag-28b exhibits a fluorescence peak at λ_ex_ = 605 nm/λ_em_ = 665 nm in PB (**a**), which is preserved upon internalization in living cells (**b**) (illustrated by the red line). Without addition of Ag-DNA variants, cell interiors exhibit a peak at λ_ex_ = 470 nm/λ_em_ = 550 nm, corresponding to autofluorescence (**c**) (illustrated by the green lines). Ag-19b shows a dominant emitter at λ_ex_ = 495 nm/λ_em_ = 560 nm in PB (**d**). Upon internalization in living cells, the peak shifts to λ_ex_ = 625 nm/λ_em_ = 680 nm (**e**) yielding a shift of Δλ_ex_ = 130 nm/Δλ_em_ = 120 nm (illustrated by the red lines). The regions of interest (ROIs) for intracellular fluorescence spectra are displayed in Figure S3. Confocal fluorescence images display the accumulation of Ag-19b at cellular membranes and inside the cell nucleus (**f**). The nucleus is labeled by GFP-histone (green) (**g**) and nuclear localization is shown by the overlay (yellow) (**h**) of the Ag-19b signal (red) (**f**) with GFP-labeled histones. The scale bar is 10 μm.

**Figure 3:**
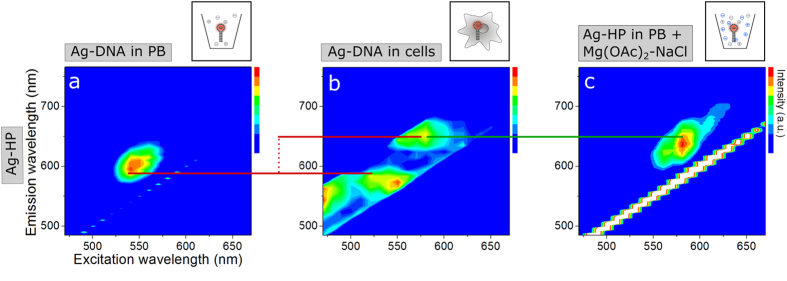
Fluorescence excitation/emission spectra of Ag-HP before (**a**) and after (**b**) internalization in *D. discoideum* wildtype cells, and in presence of specific ions (**c**). Fluorescence spectra were measured within the excitation range of 470–670 nm and the emission range of 490–760 nm, each in 10 nm steps. In PB, Ag-HP exhibits a peak at λ_ex_ = 540 nm/λ_em_ = 590 nm (**a**). Upon internalization in cells, a split into a second peak at λ_ex_ = 580 nm/λ_em_ = 650 nm occurs, yielding a shift of Δλ_ex_ = 40 nm/Δλ_em_ = 60 nm, whereas the first peak is mainly preserved (illustrated by the red lines) (**b**). Addition of 40 mM Mg(OAc)_2_ and 30 mM NaCl reproduces the red-shifted peak at λ_ex_ = 580 nm/λ_em_ = 640 nm observed only inside cells (illustrated by the green line), yet not the peak in PB (**c**). The regions of interest (ROIs) for intracellular fluorescence spectra are displayed in Figure S3.

By acquiring the excitation and emission spectra, the fluorescence properties of the Ag-DNA structures were investigated in solution and in comparison in real cell environments (Figs 2 and 3). All three types of Ag-DNA were quickly internalized into living cells and showed a homogenous distribution throughout the cytoplasm and the cell nucleus (Figure S1).

In phosphate buffer (PB), Ag-28b exhibits a single fluorescence peak at λ_ex_ = 605 nm/λ_em_ = 665 nm (Fig. 2a). When exposing these Ag-DNA structures to the interior of a living cell, the fluorescence properties of Ag-28b are not influenced, as indicated by the red line in Fig. 2a,b. The second peak at λ_ex_ = 470 nm/λ_em_ = 550 nm arises from the autofluorescence of *D. discoideum* wildtype cells (Fig. 2b,c) (indicated by the green line), as observed in all intracellular fluorescence spectra.

Shortening the DNA template reveals a significant effect on the environmental sensing function of the Ag-DNA construct. Inside cells, Ag-19b show a significant fluorescence shift of Δλ_ex_ = 130 nm/Δλ_em_ = 120 nm (Fig. 2d,e) (indicated by the red lines). This relocates the dominant peak observed in PB at λ_ex_ = 495 nm/λ_em_ = 560 nm to λ_ex_ = 625 nm/λ_em_ = 680 nm in the living cell.

A fluorescence peak in this wavelength range is typically exhibited by Ag-DNA with DNA strands longer than 19 bases, such as in Ag-28b, which stabilize larger silver clusters. Hence, these larger clusters likely require higher molecular stability to reach the fluorescent state, which can be provided by the interactions of Ag-19b with the nuclear DNA, as well as relatively dense cellular structures like membrane proteins and lipids. Accumulation of Ag-19b in these cellular regions was observed, as shown in Fig. 2f–h.

The hairpin structure (Ag-HP) was designed as a biosensor specific to intracellular ions. When in PB, Ag-HP emits at λ_ex_ = 540 nm/λ_em_ = 590 nm (Fig. 3a). Upon internalization by cells, its fluorescence properties show a split into two peaks. The previously observed peak is largely retained. Additionally, a second peak is observed at λ_ex_ = 580 nm/λ_em_ = 650 nm, thus providing a shift in emission of Δλ_em_ = 60 nm (Fig. 3a,b) (indicated by the red lines).

To confirm the role of ions in the observed red-shifted fluorescence peak, we exposed Ag-HP to various salt types and concentrations in PB. By the addition of 40 mM Mg(OAc)_2_ in combination with 30 mM NaCl (Fig. 3c), the red-shifted intensity distribution near λ_ex_ = 580 nm/λ_em_ = 650 nm, as observed inside living cells in Fig. 3b, was reproduced (Fig. 3b,c) (indicated by the green line). Exposing Ag-HPs only to monovalent (Na^+^) or only to divalent (Mg^2+^) cations did not reproduce the spectrum in Fig. 3b. Cations in particular reduce the flexibility of double stranded DNA, and can induce a conformational change, depending on the type of cations bound^43^. In high salt conditions, even chloride anions have been suggested to localize on parts of the DNA surface, as well as in the grooves^44^. We observed that the addition of chloride anions was necessary for reproducing the shift of the peak in PB to higher wavelengths, that was measured in living cells, in Mg(OAc)_2_-NaCl. Noble metal nanoparticles and clusters are known to exhibit sensitivity to their environment, like the dielectric constant of a medium, as is reflected by changes in their plasmonic resonance^39^. However, for small particles these effects typically lead to relatively small shifts in resonance^40^, which has been demonstrated recently for purified, individual Ag-DNA species, leading to an absorption shift of up to 8 nm^36^. Here, we utilize the fact that synthesis of Ag-DNA yields a heterogeneous distribution of different Ag cluster sizes and DNA conformations^45^. After synthesis, the formed silver nanoclusters can occur in a fluorescent or a dark state^45^. Hence, we attribute the observed optical shifts to rearrangements in switching these optically different emitters ‘on’ or ‘off’, resulting from conformational changes of the DNA template. This way we facilitate much larger shifts of up to Δλ_em_ = 120 nm, which allow the varying responses of Ag-19b and Ag-HP to be easily separated and identified by conventional microscopy methods. In particular, DNA hairpin structures have been shown to typically stabilize a broader range of active fluorescent emitters than the single stranded variants^46^, making them very suitable for biosensing applications.

### Tunable cytotoxicity of Ag-DNA

In addition to this biosensing functionality, we utilize the toxicity effect of endogenous silver clusters in Ag-DNA for live-cell applications. We show the tunability of cytotoxicity in Ag-DNA, which is controlled independently of the optical sensor ability of the Ag-DNA constructs.

**Figure 4:**
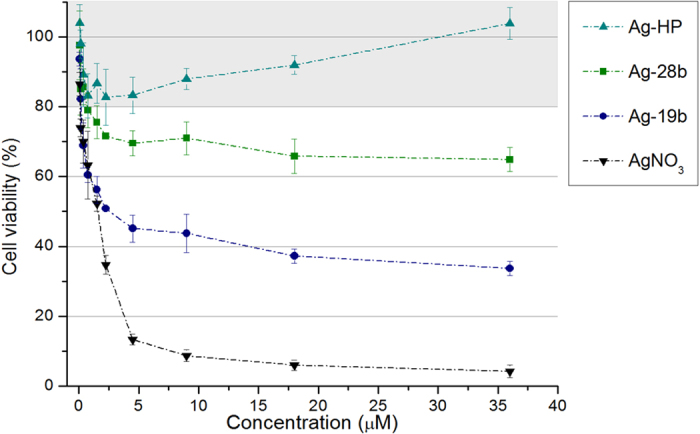
Cell viability assay of *D. discoideum* wildtype cells, after 2 h incubation with Ag-HP (▲petrol blue), Ag-28b (■ green), Ag-19b (● dark blue), and AgNO_3_ (▼ black). The number of living cells was quantified by incubation with Fluorescein diacetate and measuring the average fluorescence intensity. Cell viability of 100% was defined by a control incubated simultaneously without addition of any agents. Data shown represents the mean ± standard deviation (n = 3).

In the cytotoxicity experiment, 100% cell viability corresponds to control experiments with cells not exposed to Ag-DNA or AgNO_3_ agents under the same conditions. Figure 4 displays the concentration-dependent toxicity of Ag-28b, Ag-19b, and Ag-HP on *D. discoideum* wildtype cells, with exposures ranging between 75 nM and 36 μM. Ag-HP exhibit biocompatibility, with cell viabilities within 80–100% at all concentrations. When incubated with Ag-19b and Ag-28b, cell survival was increasingly impaired with rising Ag-DNA concentrations. For Ag-28b a slight decrease in cell viability was observed, with over ~60% viable cells at 36 μM. Ag-19b had the strongest negative effect on cell survival, still yielding ~30% of living cells at the highest concentration. As a reference measurement for cytotoxicity we used AgNO_3_, which readily releases free Ag(I)ions in solution. This resulted in the highest cytotoxic effect for the same concentration of silver, as compared to the DNA-stabilized silver clusters.

To exclude other sources for cytotoxicity beside Ag-DNA, the following steps were taken. We removed potentially harmful byproducts or educts possibly causing toxicity by filtration of the synthesized Ag-DNA. Also we confirmed experimentally that a toxic effect caused by oligonucleotides is unlikely. Furthermore, for the cytotoxicity experiments, incubation steps and subsequent cell viability assays were performed in the dark, to exclude possible light-induced toxicity, like ROS generation. These points allow us to identify the silver in the Ag-DNA constructs as the only significant source of cytotoxicity, which is supported by the biocompatibility of Ag-HP.

We conclude from these results that the DNA templates can protect cells from silver and the selected DNA-sequence determines the toxic effect, ranging from non-toxic for Ag-HP to highly toxic for Ag-19b. This mechanism of protection results from distinct interactions of silver with DNA bases and the steric conditions of the Ag-DNA, varying the degree of silver exposure to cellular structures. Due to high binding energies of silver clusters to cytosine and guanine bases, which are several times stronger than Watson-Crick base pairs^46^, we can assume that the silver clusters mainly remain contained in the Ag-DNA structures. Hence, the observed cytotoxicity effect of single stranded Ag-DNA nanomaterials most likely results from direct contact of cellular structures and molecules with exposed areas of the silver clusters as well as possibly released individual Ag(I)ions.

In Ag-HP, the silver cluster is surrounded by a Poly-C loop. As guanine and cytosine bases in particular show a high affinity to Ag(I)ions^45^,^47^,48, multiple Ag(I)ions-DNA base connections are formed which lead to a strongly shielded silver cluster, allowing for a decrease in Ag-DNA cytotoxicity. Both Ag-28b and Ag-19b constructs are single stranded DNA templates, which are expected to wrap around the silver cluster upon reduction with NaBH_4_, due to Ag(I)ions-base bonding. The sequences in Ag-28b and Ag-19b lack the high density of cytosines of the Poly-C loop in Ag-HP, hence a larger area of the silver nanocluster is exposed in Ag-28b and Ag-19b, which can interact with surrounding molecules and impact cellular processes. Since Ag-28b provides a higher amount of cytosine and guanine bases, and additionally, its longer strand offers the possibility of a more stable Ag-DNA conformation, it yields a lower cytotoxicity than its shorter counterpart Ag-19b. In these three variants, we show the potential for cytotoxicity tuning by adapting the cytosine and guanine amount and location in the implemented DNA template.

## Conclusion

In conclusion, we show that the unique properties of Ag-DNA allow for nanomaterials with multiple functionalities, targeted at optical sensing in combination with tunable cytotoxicity, by simple modifications of Ag-DNA designs. Ag-28b exhibits stable fluorescence properties and is thus suitable for bright and persistent labeling of cells, as well as localization experiments. Its shorter counterpart, Ag-19b, is environmentally sensitive with an optical emission shift of Δλ_em_ = 120 nm and exhibits higher cytotoxicity, making it interesting as an anti-bacterial or anti-cancer agent. The biocompatible, non-toxic Ag-HP optically responds to intracellular salt concentrations, visible as a large fluorescence shift of about Δλ_em_ = 60 nm, which allows a convenient detection of signal changes for both, Ag-HP and Ag-19b, by commonly used fluorescence microscope systems.

These results will allow us to extend these Ag-DNA constructs to more complex DNA-origami structures, and the investigations from single cells to living organisms. Therefore, Ag-DNA represent a new nanomaterial with high potential for a multi-functional nanoagent, combining bio-sensing and effector functionality. Possible targeted DNA-sequence specific applications would open a large field of patient-specific treatment including cancer or rare and degenerative diseases, as well as specific antimicrobial targeting.

## Methods

### Chemicals

DNA strands 5′-CAC CGC TTT TGC CTT TTG GGG ACG GAT A-3′ (28b), 5′-TGC CTT TTG GGG ACG GAT A-3′ (19b), and (b) 5′-CT TAC CTC CCC CCC CCC CCA GGT AAG-3′ (HP) with standard desalting (Integrated DNA Technologies, USA), AgNO_3_ (99.9999%, Sigma Aldrich, The Netherlands), NH_4_OAc (99.99%, Sigma Aldrich, The Netherlands) NaBH_4_ (99%, Sigma Aldrich, The Netherlands), KH_2_PO_4_ (>99%, Sigma Aldrich, The Netherlands), Na_2_HP, O_4_ (>99%, Sigma Aldrich, The Netherlands), Maltose (Duchefa Biochemie B. V., Netherlands), Fluorescein diacetate (F7378, Sigma Aldrich Merck, Germany), HL5-C medium (Formedium™, United Kingdom), NaCl (Fisher Scientific Company LLC, USA), Mg(OAc)_2_ (>99%, Sigma Aldrich, The Netherlands), Geneticin (Sigma Aldrich, The Netherlands), 3 kDa and 50 kDa centrifugal filter (Merck Millipore, Germany) were used as received without further purification. Bi-distilled water from a Millipore system (Milli-Q Academic A-10, Merck Millipore Germany) was used for all solutions.

### Ag-DNA preparation

For synthesis of (a) Ag-28b and Ag19b, and (b) Ag-HP solutions, 50 nmol of the respective DNA molecules were mixed with AgNO_3_ in a ratio of (a) 9.6:1 silver ions:DNA strands, or (b) 7:1 silver ions:DNA strands in 1 ml 20 mM NH_4_OAc. The solutions were incubated for 30 min at 4 °C and then reduced with NaBH_4_ at 4 °C over night. The reduction ratios were (a) 0.5:1 and (b) 0.3:1 NaBH_4_:Ag^+^. To exclude formed silver clumps and unbound Ag(I) ions as well as other salts, Ag-DNA constructs were filtered using a 3 kDa centrifugal filter and subsequently a 50 kDa centrifugal filter, simultaneously exchanging the buffer with phosphate buffer (PB; 3.57 mM KH_2_PO_4_, 3.46 mM Na_2_HPO_4_, pH 6.0). The concentration of the DNA was calculated based on the absorption at 260 nm, measured with a Cary 50 UV-Vis spectrophotometer (Varian Medical Systems, Netherlands). For the reproduction of the observed peaks, various concentrations of Mg(OAc)_2_ and NaCl were added to a 15 μM solution of Ag-HP in PB and the fluorescence properties measured. The excitation and emission properties of the Ag-DNA in PB were determined by Cary Eclipse fluorimeter (Varian Medical Systems, Netherlands).

### Cell culture

*Dictyostelium discoideum* HG1729 histone-GFP in AX2 and *Dictyostelium discoideum* Wildtype in AX2 cells were cultured in HL5-C medium adjusted to pH 6.7 at 21 °C; medium for HG1729 histone-GFP was supplemented with 10 μg/ml Geneticin. For the experiments the cells were harvested below a confluency of 40% and washed three times with PB by centrifugation at 400 g for 5 min. Subsequently, cells dispersed in PB were seeded onto an observation chamber (composed of a cover glass and a custom-made Teflon® frame) and rested for 30 min, allowing the cells to settle down.

### Microscopy

Imaging of cells incubated with Ag-DNA was performed by confocal microscopy set-up equipped with a Yokogawa 10,000 rpm spinning disc unit (Andor Technology Ltd., UK), Eclipse TiE Nikon inverted microscope (Nikon Corporation, Japan) and an IXON Ultra EMCCD camera (Andor Technology Ltd., UK). Ag-28b and Ag-19b were added to *D. discoideum* HG1729 histone-GFP cells to an end concentration of 4.5 μM and 3 μM, respectively, in an observation chamber and immediately imaged with a 100x oil immersion objective with NA 1.45. Ag-28b and Ag-19b were excited at 640 nm, histone GFP at 488 nm. As negative controls, also cells without Ag-DNA were imaged, as well as cells incubated in 36 μM AgNO_3_ and 4.5 μM 28b-DNA, separately and mixed. Local fluorescence spectra measurements were performed with a Leica TCS SP8 STED 3X Microscope and with a 63x objective lens (Leica Microsystems, Germany), additionally equipped with HyD detectors (Leica Microsystems, Germany). The excitation wavelengths ranged from 470 to 670 nm in 10 nm steps and the emission wavelengths ranged from 480 to 770 nm, with a step size of 10 nm and a detection window of 10 nm. For this assay, solutions of Ag-28b (4.5 μM), Ag-19b (3 μM) and Ag-HP (15 μM) were incubated with *D. discoideum* wildtype cells.

### Cytotoxicity assay

*D. discoideum* wildtype cells were harvested and washed three times with a maltose-loaded phosphate buffer (3.57 mM KH_2_PO_4_, 3.46 mM Na_2_HPO_4_, 52.58 mM Maltose, pH 6.70) by centrifugation at 400 g for 4 min. Cells were incubated for 2 h with Ag-28b, Ag-19b and Ag-HP, with concentrations ranging between 75 nM and 36 μM, as triplicates with 2.5 × 10^4^ cells/condition in the dark. As a positive control, incubation with AgNO_3_ was performed under the same conditions, as well as a negative control without agent incubation. To determine cell viability, cells were incubated with 50 μg/ml Fluorescein diacetate for 15 min, and the average fluorescence intensity was measured using a TECAN Infinite M200 well plate reader (Tecan Trading AG, Switzerland). To avoid background fluorescence from the Ag-DNA, 50 mM NaCl was introduced to all samples prior to measurements.

## Additional Information

**How to cite this article**: Bossert, N. *et al*. Fluorescence-tunable Ag-DNA biosensor with tailored cytotoxicity for live-cell applications. *Sci. Rep.* **6**, 37897; doi: 10.1038/srep37897 (2016).

**Publisher's note:** Springer Nature remains neutral with regard to jurisdictional claims in published maps and institutional affiliations.

## Supplementary Material

Supplementary Information

## References

[b1] Sharma J. . Silver Nanocluster Aptamers: *In Situ* Generation of Intrinsically Fluorescent Recognition Ligands for Protein Detection. Chem. Commun. 47, 2294 (2011). 10.1039/c0cc03711g 21152540

[b2] Sengupta B. . DNA Templates for Fluorescent Silver Clusters and I-Motif Folding. J. Phys. Chem. 113, 19518 (2009).

[b3] Richards C. I. . Oligonucleotide-Stabilized Ag Nanocluster Fluorophores. J. Am. Chem. Soc. 130, 5038 (2008). 18345630 10.1021/ja8005644 PMC2766658

[b4] Vosch T., Antoku Y., Hsiang J.-C., Richards C. I., Gonzalez J. I. & Dickson R. M. Strongly Emissive Individual DNA-Encapsulated Ag Nanoclusters as Single-Molecule Fluorophores. Proc. Natl. Acad. Sci. USA. 104, 12616 (2007). 17519337 10.1073/pnas.0610677104 PMC1937515

[b5] Schultz D. . Dual-color Nanoscale Assemblies of Structurally Stable, Few-Atom Silver Clusters, as Reported by Fluorescence Resonance Energy Transfer. ACS Nano. 7, 9798 (2007). 10.1021/nn4033097 24090435

[b6] Sengupta B., Ritchie C. M., Buckman J. G., Johnsen K. R., Goodwin P. M. & Petty J. T. Base-Directed Formation of Fluorescent Silver Clusters. J. Phys. Chem. 112, 18776 (2008). 10.1021/jp804031v PMC6178949 30319723

[b7] Driehorst T., O’Neill P., Goodwin P. M., Pennathur S. & Fygenson D. K. Distinct Conformations of DNA-Stabilized Fluorescent Silver Nanoclusters Revealed by Electrophoretic Mobility and Diffusivity Measurements. Langmuir. 27, 8923 (2001). 10.1021/la200837z 21682258

[b8] Yeh H.-C., Sharma J., Han J. J., Martinez J. S. & Werner J. H. A DNA-Silver Nanocluster Probe That Fluoresces upon Hybridization. Nano Letters. 10, 3106 (2010). 20698624 10.1021/nl101773c

[b9] Yeh H.-C., Sharma J., Shih I.-M., Vu D. M., Martinez J. S. & Werner J. H. A. A Fluorescence Light-Up Ag Nanocluster Probe That Discriminates Single-Nucleotide Variants by Emission Color. J. Am. Chem. Soc. 134, 11550 (2012). 22775452 10.1021/ja3024737 PMC3593641

[b10] Guo W., Yuan J., Dong Q. & Wang E. Highly Sequence-Dependent Formation of Fluorescent Silver Nanoclusters in Hybridized DNA Duplexes for Single Nucleotide Mutation Identification. J. Am. Chem. Soc. 132, 932 (2010). 20038102 10.1021/ja907075s

[b11] Liu J.-J., Song X.-R., Wang Y.-W., Zheng A.-X., Chen G.-N. & Yang H.-H. Label-Free and Fluorescence Turn-On Aptasensor for Protein Detection via Target-Induced Silver Nanoclusters Formation. Anal. Chim. Acta, 749, 70 (2012). 23036469 10.1016/j.aca.2012.09.002

[b12] Arcizet D., Meier B., Sackmann E., Radler J. O. & Heinrich D. Temporal Analysis of Active and Passive Transport in Living Cells. Phys. Rev. Lett. 101, 248103 (2008). 19113674 10.1103/PhysRevLett.101.248103

[b13] Götz M., Hodeck K. F., Witzel P., Nandi A., Lindner B. & Heinrich D. Probing Cytoskeleton Dynamics by Intracellular Particle Transport Analysis. Eur. Phys. J. Spec. Top. 224, 1169 (2015).

[b14] Otten M., Nandi A., Arcizet D., Gorelashvili M., Lindner B. & Heinrich D. Local Motion Analysis Reveals Impact of the Dynamic Cytoskeleton on Intracellular Subdiffusion. Biophys. J. 102, 758 (2012). 22385846 10.1016/j.bpj.2011.12.057 PMC3283776

[b15] Pelzl C., Arcizet D., Piontek G., Schlegel J. & Heinrich D. Axonal Guidance by Surface Microstructuring for Intracellular Transport Investigations. Chemphyschem. 10, 2884 (2009). 19760697 10.1002/cphc.200900555

[b16] Sharma J. . A DNA-Templated Fluorescent Silver Nanocluster with Enhanced Stability. Nanoscale. 4, 4107 (2012). 22648534 10.1039/c2nr30662j

[b17] Kuehl R. . Preventing Implant-Associated Infections by Silver Coating. Antimicrob. Agents Chemother. 60, 2467 (2016). 26883700 10.1128/AAC.02934-15 PMC4808148

[b18] Asghari S., Logsetty S. & Liu S. Imparting Commercial Antimicrobial Dressings with Low-Adherence to Burn Wounds. Burns. 42, 877 (2016). 26847614 10.1016/j.burns.2016.01.005

[b19] Halstead F. D. . Antimicrobial Dressings: Comparison of the Ability of a Panel of Dressings to Prevent Biofilm Formation by Key Burn Wound Pathogens. Burns. 41, 1683 (2015). 26188884 10.1016/j.burns.2015.06.005

[b20] Chernousova S. & Epple M. Silver as Antibacterial Agent: Ion, Nanoparticle, and Metal. Angew. Chem. Int. Ed. 52, 1636 (2013). 10.1002/anie.201205923 23255416

[b21] Kedziora A., Korzekwa K., Strek W., Pawlak A., Doroszkiewicz W. & Bugla-Ploskonska G. Silver Nanoforms as a Therapeutic Agent for Killing *Escherichia Coli* and Certain ESKAPE Pathogens. Curr. Microbiol. 73, 139 (2016). 27086305 10.1007/s00284-016-1034-8 PMC4899487

[b22] Radzig M. A., Nadtochenko V. A., Koksharova O. A., Kiwi J., Lipasova V. A. & Khmel I. A. Antibacterial Effects of Silver Nanoparticles on Gram-Negative Bacteria: Influence on the Growth and Biofilms Formation, Mechanisms of Action. Colloids Surf. B. 102, 300 (2016). 10.1016/j.colsurfb.2012.07.039 23006569

[b23] Mussa Farkhani S., Asoudeh Fard A., Zakeri-Milani P., Shahbazi Mojarrad J. & Valizadeh H. Enhancing Antitumor Activity of Silver Nanoparticles by Modification with Cell-Penetrating Peptides. Artif. Cells Nanomed. Biotechnol. 1–7 [Epub ahead of print]. doi: 10.1080/21691401.2016.1200059 (2016). 10.1080/21691401.2016.1200059 27357085

[b24] Sanpui P., Chattopadhyay A. & Ghosh S. S. Induction of Apoptosis in Cancer Cells at Low Silver Nanoparticle Concentrations Using Chitosan Nanocarrier. ACS Appl. Mater Interfaces. 3, 218 (2011). 21280584 10.1021/am100840c

[b25] Pandurangan M. . Time and Concentration-Dependent Therapeutic Potential of Silver Nanoparticles in Cervical Carcinoma Cells. Biol. Trace Elem. Res. 170, 309 (2016). 26276565 10.1007/s12011-015-0467-4

[b26] Mao B.-H., Tsai J.-C., Chen C.-W., Yan S.-J. & Wang Y.-J. Mechanisms of Silver Nanoparticle-Induced Toxicity and Important Role of Autophagy. Nanotoxicology. 10, 1021 (2016). 27240148 10.1080/17435390.2016.1189614

[b27] Li Y. . Differential Genotoxicity Mechanisms of Silver Nanoparticles and Silver Ions. Arch. Toxicol. [Epub ahead of print]. doi: 10.1007/s00204-016-1730-y (2016). 10.1007/s00204-016-1730-y 27180073

[b28] Frohlich E. E. & Frohlich E. Cytotoxicity of Nanoparticles Contained in Food on Intestinal Cells and the Gut Microbiota. Int. J. Mol. Sci. 17 (2016). 10.3390/ijms17040509 PMC4848965 27058534

[b29] Georgantzopoulou A. Effects of Silver Nanoparticles and Ions on a Co-Culture Model for the Gastrointestinal Epithelium. Part. Fibre Toxicol. 13, 9 (2016). 26888332 10.1186/s12989-016-0117-9 PMC4756536

[b30] Vrcek I. V. . Comparison of *In Vitro* Toxicity of Silver Ions and Silver Nanoparticles on Human Hepatoma Cells. Environ. Toxicol. 31, 679 (2016). 25448069 10.1002/tox.22081

[b31] Yin J. . One-Step Engineering of Silver Nanoclusters-Aptamer Assemblies as Luminescent Labels to Target Tumor Cells. Nanoscale. 4, 110 (2012). 22080331 10.1039/c1nr11265a

[b32] Sun Z. . Ag Cluster-Aptamer Hybrid: Specifically Marking the Nucleus of Live Cells. Chem. Commun. 47, 11960 (2011). 10.1039/c1cc14652a 21959749

[b33] Yu J., Choi S., Richards C. I., Antoku Y. & Dickson R. M. Binding-Induced Fluorescence Turn-On Assay Using Aptamer-Functionalized Silver Nanocluster DNA Probes. Photochem. Photobiol. Sci. 84, 1435 (2008). 10.1111/j.1751-1097.2008.00434.x PMC2600602 18764887

[b34] Li J., Zhong X., Zhang H., Le X. C. & Zhu J.-J. Binding-Induced Fluorescence Turn-On Assay Using Aptamer-Functionalized Silver Nanocluster DNA Probes. Anal. Chem. 84, 5170 (2012). 22607314 10.1021/ac3006268

[b35] Schultz D. . Evidence for Rod-Shaped DNA-Stabilized Silver Nanocluster Emitters. Adv. Mater. 25, 2797 (2013). 23371742 10.1002/adma.201204624

[b36] Copp S. M. . Cluster Plasmonics: Dielectric and Shape Effects on DNA-Stabilized Silver Clusters. J. Phys. Chem. Lett. 5, 959 (2014). 27187492 10.1021/acs.nanolett.6b00723

[b37] O’Neill P. R., Velazquez L. R., Dunn D. G., Gwinn E. G. & Fygenson D. K. Hairpins with Poly-C Loops Stabilize Four Types of Fluorescent Ag_n_: DNA. J. Phys. Chem. C. 113, 4229 (2009).

[b38] Kelly K. L., Coronado E., Zhao L. L. & Schatz G. C. The Optical Properties of Metal Nanoparticles: The Influence of Size, Shape, and Dielectric Environment. J. Phys. Chem. B 107, 668 (2003).

[b39] Chen H., Kou X., Yang Z., Ni W. & Wang J. Shape- and Size-Dependent Refractive Index Sensitivity of Gold Nanoparticles. Langmuir. 24, 5233 (2008). 18435552 10.1021/la800305j

[b40] Schultz D. & Gwinn E. Stabilization of Fluorescent Silver Clusters by RNA Homopolymers and Their DNA Analogs: C,G Versus A,T(U) Dichotomy. Chem. Commun. 47, 4715 (2011). 10.1039/c0cc05061j 21412565

[b41] Denisov V. P. & Halle B. Sequence-Specific Binding of Counterions to B-DNA. Proc. Natl. Acad. Sci. USA. 97, 629 (2000). 10639130 10.1073/pnas.97.2.629 PMC15381

[b42] Varnai P. & Zakrzewska K. DNA and its Counterions: a Molecular Dynamics Study. Nucleic Acids Res. 32, 4269 (2004). 15304564 10.1093/nar/gkh765 PMC514386

[b43] Liubysh O. O., Vlasiuk A. V. & Perepelytsya S. M. Structuring of Counterions Around DNA Double Helix: a Molecular Dynamics Study. Ukr. J. Phys. 60, 433 (2015).

[b44] Schultz D. & Gwinn E. G. Silver Atom and Strand Numbers in Fluorescent and Dark Ag:DNAs. Chem. Commun. 48, 5748 (2012). 10.1039/c2cc17675k 22552272

[b45] Gwinn E. G., O’Neill P., Guerrero A. J., Bouwmeester D. & Fygenson D. K. Silver Atom and Strand Numbers in Fluorescent and Dark Ag:DNAs. Adv. Mater. 20, 279 (2008). 10.1039/c2cc17675k 22552272

[b46] Swasey S. M., Leal L. E., Lopez-Acevedo O., Pavlovich J. & Gwinn E. G. Silver (I) as DNA Glue: Ag(+)-Mediated Guanine Pairing Revealed by Removing Watson-Crick Constraints. Sci. Rep. 5, 10163 (2015). 25973536 10.1038/srep10163 PMC4431418

[b47] Torigoe H., Okamoto I., Dairaku T., Tanaka Y., Ono A. & Kozasa T. Thermodynamic and Structural Properties of the Specific Binding between Ag(+) Ion and C:C Mismatched Base Pair in Duplex DNA to Form C-Ag-C Metal-Mediated Base Pair. Biochemie. 94, 2431 (2012). 10.1016/j.biochi.2012.06.024 22766014

