# Corrigendum: Fluorescence-tunable Ag-DNA biosensor with tailored cytotoxicity for live-cell applications

**DOI:** 10.1038/srep45882

**Published:** 2017-04-12

**Authors:** Nelli Bossert, Donny de Bruin, Maria Götz, Dirk Bouwmeester, Doris Heinrich

Scientific Reports 6: Article number: 3789710.1038/srep37897; published online: 11 30 2016; updated: 04 12 2017

In the ‘Introduction’ and ‘Results and Discussion’ sections of this manuscript, the authors used 2 sequences that were previously published and cited already as references 35 and 44. To better reflect the use of these sequences within the Introduction:

“Using these dependencies, we designed Ag-DNA templates to function as an optical sensor in living cells’ interior environments (Fig. 1b–d) (for DNA sequences please see Table S1)”.

Now reads:

“Using these dependencies, we selected and optimized Ag-DNA templates to function as an optical sensor in living cells’ interior environments (Fig. 1b–d) (for DNA sequences please see Table S1)”.

In the Results and Discussion section under the ‘Ag-DNA nanoagent design’ subheading:

“The design of DNA sequences is the result of optimizing fluorescence and/or sensor properties of Ag-DNA within the cell by varying the base sequences for enhanced performance”.

Now reads:

“The choice of DNA sequences is the result of optimizing fluorescence and/or sensor properties of Ag-DNA within the cell by varying the base sequences for enhanced performance”.

Secondly,

“The DNA template, consisting of 28 single bases, is designed to yield an Ag-DNA construct (Ag-28b in Fig. 1b) with strong and stable fluorescence properties upon insertion into living cells”.

Now reads:

“The DNA template, consisting of 28 single bases, yields an Ag-DNA construct (Ag-28b in Fig. 1b) with strong and stable fluorescence properties^35^, which we show are retained upon insertion into living cells”.

In addition,

“Here, we demonstrate that by a simple modification of Ag-28b, we produced an Ag-DNA construct which shows optical sensor functionality inside living cells: shortening the 28b sequence by nine bases produces the Ag-19b construct (Fig. 1c), which exhibits an optical response to the intracellular environment”.

Now reads:

“Here, we demonstrate that a simple modification of Ag-28b produces an Ag-DNA construct which shows optical sensor functionality inside living cells: shortening the 28b sequence by nine bases produces the Ag-19b construct^44^ (Fig. 1c), which exhibits an optical response to the intracellular environment”.

Finally,

“To decrease the toxicity level to a biocompatible Ag-DNA for an optical biosensor sensitive to intracellular ions, we designed an Ag-hairpin (Ag-HP) construct (Fig. 1d)”.

Now reads:

“To decrease the toxicity level to a biocompatible Ag-DNA for an optical biosensor sensitive to intracellular ions, we designed an Ag-hairpin (Ag-HP) construct (Fig. 1d), as DNA hairpins with Poly-C loops have been shown to stabilize strongly fluorescent Ag-DNA species^37^”.

In addition, the original Article contained typographical errors in the reference list. These errors have now been corrected in the HTML and PDF versions of this Article.

