# Supplementary material for: Fluorescence-tunable Ag-DNA biosensor with tailored cytotoxicity for live-cell applications
Source: Sci Rep. 2016 Nov 30;6:37897. doi: 10.1038/srep37897 (PMC5129012; doi:10.1038/srep37897)
Supplement: Supplementary Information [file srep37897-s1.pdf]

Supporting Information

Fluorescence-tunable Ag-DNA biosensor with tailored cytotoxicity for live-cell applications

Nelli Bossert, Donny de Bruin, Maria Götz, Dirk Bouwmeester, Doris Heinrich[*]

Supplemental Data

Table S1. DNA sequences used for Ag-DNA synthesis.

DNA sequence	Abbreviation
5'-CAC CGC TTT TGC CTT TTG GGG ACG GAT A-3'	28b
5'-TGC CTT TTG GGG ACG GAT A-3'	19b
5'- CT TAC CTC CCC CCC CCC CCA GGT AAG-3'	HP

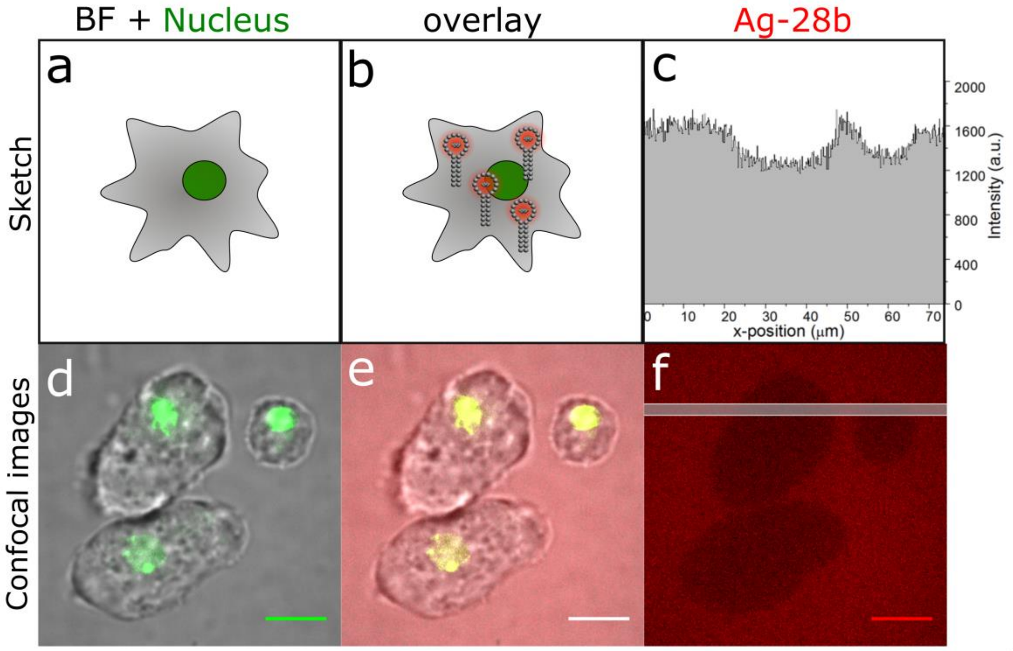

Figure S1. Internalization and distribution of Ag-28b inside *D. discoideum* cells. Overlay of brightfield images of cells with labeled GFP histones (green), showing the cell nucleus (a, d) (excitation at 488 nm), and including Ag-28b fluorescence (b, e) (excitation at 640 nm). Profile of the mean fluorescence intensity of Ag-28b (c) for positions along the grey line in the image of red Ag-28b fluorescence (f), showing ~80% of the intensity from the surrounding medium at positions corresponding to the cell interior. The scale bars are 10 μm.

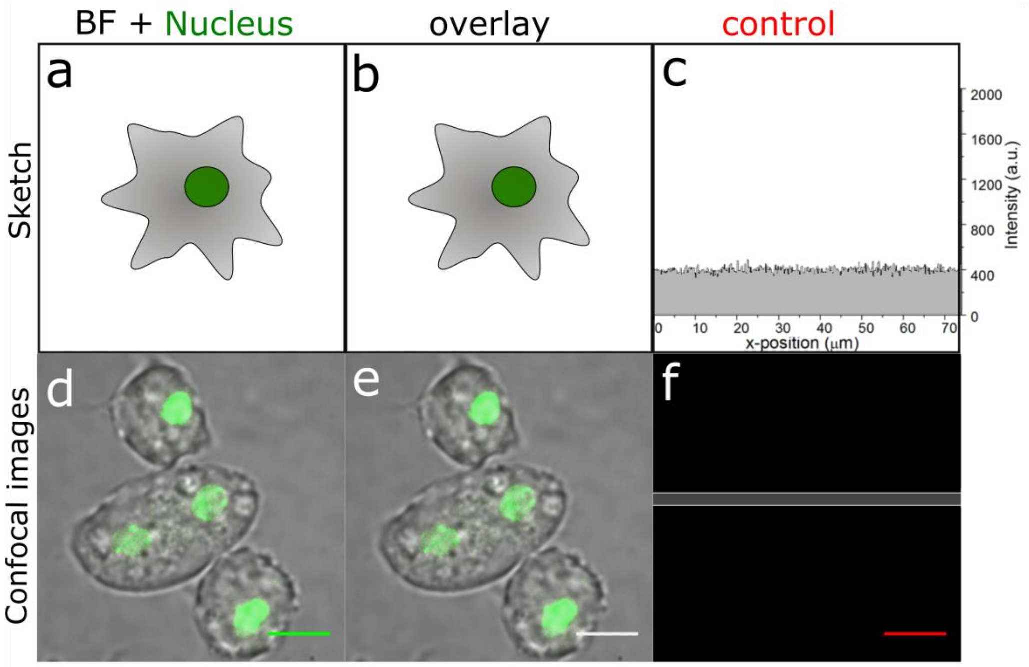

Figure S2. Negative control for the internalization and distribution of Ag-28b inside *D. discoideum* cells in Figure S1. Overlay of brightfield images of cells with labeled GFP histones, showing the cell nucleus (a, d) (excitation at 488 nm), and including any red autofluorescence (b, e) (excitation at 640 nm). Profile of the mean fluorescence intensity at 640 nm excitation (c) for positions along the grey line in the image of only the red channel (f). The scale bars are 10μm.

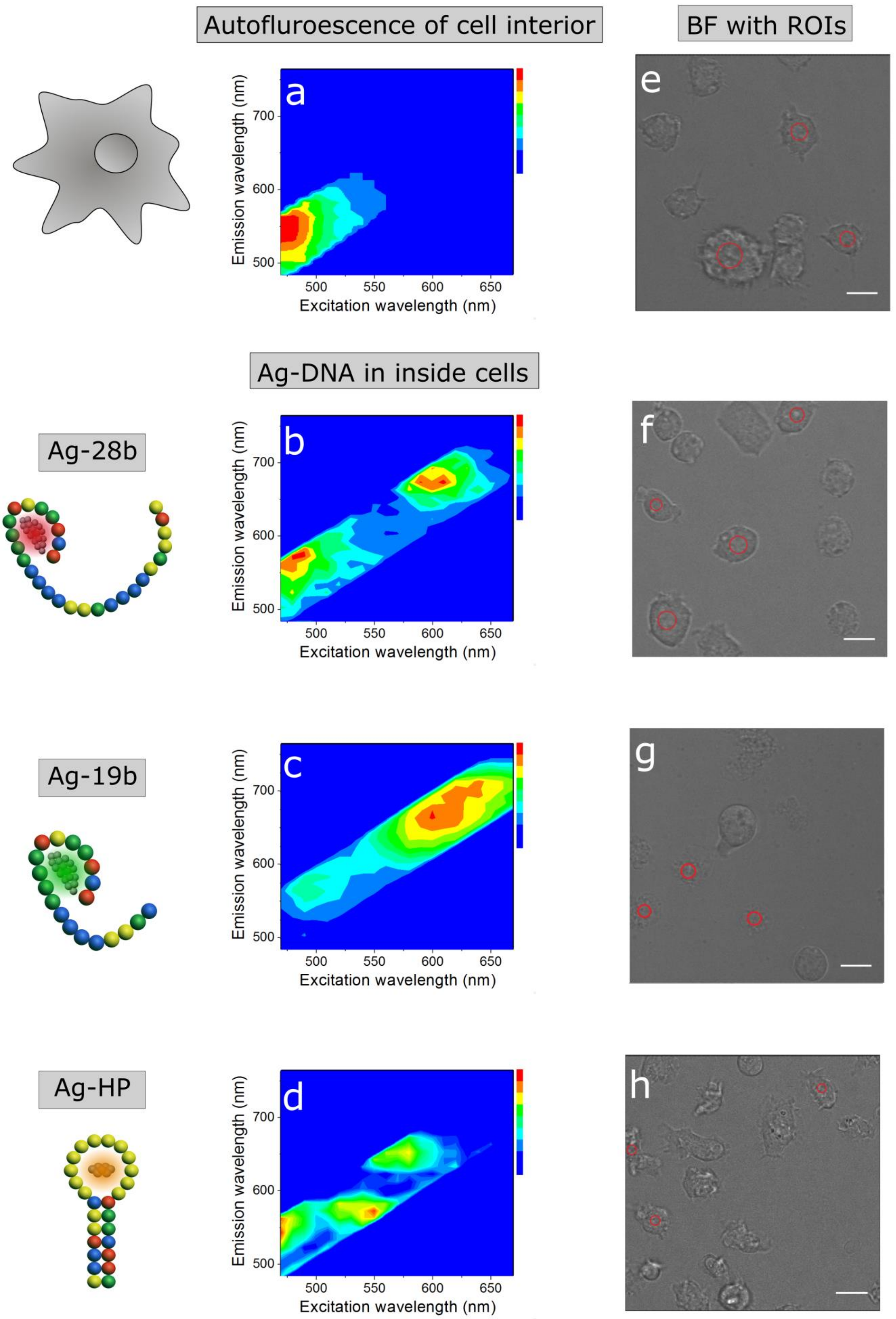

Autofluroescence of cell interior
BF with ROIs
Ag-DNA in inside cells
Ag-28b
Ag-19b
Ag-HP
a
b
c
d
e
f
g
h
Emission wavelength (nm)
Excitation wavelength (nm)
500
550
600
650
700

Figure S3. Region of interest (ROI) selection (e-h) for the fluorescence spectra analysis of Ag-DNA variants after internalization in *D. discoideum* wildtype cells (a-d). The 1st column shows sketches of used Ag-DNAs. DNA sequence color code: Cytosine = yellow, Guanine = green, Thymine = blue, Adenine = red. The spectra correspond to the fluorescence collected from the ROIs designated by red circles in the microscopy images. Fluorescence spectra were measured within excitation range of 470-670 nm and emission range of 490-760 nm, each in 10 nm steps. ROIs were chosen to remain entirely within the center of the cell throughout the measurement to avoid collection of light from the surrounding medium. The scale bars are 10μm.
